# Mitophagy promotes sorafenib resistance through hypoxia-inducible ATAD3A dependent Axis

**DOI:** 10.1186/s13046-020-01768-8

**Published:** 2020-12-07

**Authors:** Hong Wu, Tao Wang, Yiqiang Liu, Xin Li, Senlin Xu, Changtao Wu, Hongbo Zou, Mianfu Cao, Guoxiang Jin, Jinyi Lang, Bin Wang, Baohua Liu, Xiaolin Luo, Chuan Xu

**Affiliations:** 1 Guangdong Key Laboratory of Genome Stability and Human Disease Prevention, Shenzhen University Health Science Center, 518055 Shenzhen, China; 2 Integrative Cancer Center&Cancer Clinical Research Center, Sichuan Cancer Hospital & Institute Sichuan Cancer Center, School of Medicine, University of Electronic Science and Technology of China, Chengdu, 610000 P. R. China; 3 Department of Experimental Research, The Affiliated Tumor Hospital of Guangxi Medical University, Nanning, 510000 P. R. China; 4 Department of Gastroenterology, Daping Hospital, Army Medical University (Third Military Medical University), Chongqing, 400042 P. R. China; 5 Institute of Pathology and Southwest Cancer Center, Southwest Hospital and Key Laboratory of Tumor Immunopathology, Army Medical University (Third Military Medical University), Chongqing, 400042 P. R. China

**Keywords:** Mitophagy, ATAD3A, Hypoxia, Sorafenib resistance

## Abstract

**Background:**

The identification of novel targets for recovering sorafenib resistance is pivotal for Hepatocellular carcinoma (HCC) patients. Mitophagy is the programmed degradation of mitochondria, and is likely involved in drug resistance of cancer cells. Here, we identified hyperactivated mitophagy is essential for sorafenib resistance, and the mitophagy core regulator gene ATAD3A (ATPase family AAA domain containing 3A) was down regulated in hypoxia induced resistant HCC cells. Blocking mitophagy may restore the sorafenib sensitivity of these cells and provide a new treatment strategy for HCC patients.

**Methods:**

Hypoxia induced sorafenib resistant cancer cells were established by culturing under 1% O_2_ with increasing drug treatment. RNA sequencing was conducted in transfecting LM3 cells with sh-ATAD3A lentivirus. Subsequent mechanistic studies were performed in HCC cell lines by manipulating ATAD3A expression isogenically where we evaluated drug sensitivity, molecular signaling events. In vivo study, we investigated the combined treatment effect of sorafenib and miR-210-5P antagomir.

**Results:**

We found a hyperactivated mitophagy regulating by ATAD3A-PINK1/PARKIN axis in hypoxia induced sorafenib resistant HCC cells. Gain- and loss- of ATAD3A were related to hypoxia-induced mitophagy and sorafenib resistance. In addition, ATAD3A is a functional target of miR-210-5p and its oncogenic functions are likely mediated by increased miR-210-5P expression. miR-210-5P was upregulated under hypoxia and participated in regulating sorafenib resistance. In vivo xenograft assay showed that miR-210-5P antagomir combined with sorafenib abrogated the tumorigenic effect of ATAD3A down-regulation in mice.

**Conclusions:**

Loss of ATAD3A hyperactivates mitophagy which is a core event in hypoxia induced sorafenib resistance in HCC cells. Targeting miR-210-5P-ATAD3A axis is a novel therapeutic target for sorafenib-resistant HCC.

**Supplementary Information:**

The online version contains supplementary material available at 10.1186/s13046-020-01768-8.

## Background

Mitophagy is a mitochondrial form of autophagy that is critical for mitochondrial quality control and homeostasis [1, 2]. In response to various stimuli, mitochondria undergo self-depolarization and the damaged organelles are identified by autophagosomes which then fuse with lysosomes to complete the degradation process [3, 4]. Defective or excessive mitophagy is a pathological factor in chronic diseases including parkinson’s disease (PD), diabetes, myocardial ischemia reperfusion (MIR) injury, ankylosing spondylitis (AS) and cancers [5]. Several mitophagic pathways have been identified that counteract therapy-induced mitochondrial damage [5, 6] . Guo et al. reported that reversing mitophagy inhibition by the PIK3CA/AKT1/MTOR /RPS6KB1 pathway reduced the risk of colorectal cancer (CRC) development [7]. Katreddy et al. found that EGFR down-regulation cleared ovarian cancer cells by activating the mitophagy/mTORC2/Akt axis [8]. Furthermore, mitophagy suppression via knocking down key mitophagy receptors such as PINK1, FUNDC1 or AMBRA1 could chemo-sensitize cancer cells [9, 10]. The E3 ubiquitin-ligase ARIH1 and not Parkin mediates PINK1-induced mitophagic responses to cisplatin/etoposide in breast and lung adenocarcinomas [11]. In fact, the predominance of certain mitophagy receptors or mediators in specific cancer subtypes is a decisive factor in therapeutic resistance via mitochondrial clearance [6, 12], and the underlying mechanisms are still unclear.

The ubiquitin-dependent PINK1/Parkin pathway is the most widely studied mammalian mitophagy cascade. PINK1 is an outer mitochondrial membrane (OMM) Ser/Thr kinase that is stabilized upon mitochondrial depolarization, and induces Parkin E3 activity and recruits Parkin by phosphorylating it at Ser-65 [13]. Recent studies has shown that ectopic expression of PINK1 plays a dual role in cancer development and drug treatment [14]. Wang et al. reported that the induction of PINK1/Parkin-mediated mitophagy sensitized tongue cancer cells to ZnO NPs [15]. In contrast, Villa et al. showed that ARIH1/HHARI triggered PINK1-dependent mitophagy in the breast and lung adenocarcinomas and protected them from chemotherapy-induced death [11]. In the recent study, our group showed that the AAA domain containing 3A (ATAD3A) protein could prevent excessive accumulation of PINK1 and therefore inhibited unnecessary mitophagy in stem and progenitor cells [16]. As an up-regulator of PINK1, ATAD3A spans the inner mitochondrial membrane with its two terminal domains in the outer membrane and the matrix [17]. However, little is known regarding its role in cancer development and drug sensitivity under hypoxia microenvironment of solid tumors.

microRNAs are members of a large class of endogenous small noncoding RNAs that control gene expression and regulate a wide array of biological processes by binding to the 3′-untranslated regions (3′-UTR) directly to regulate target mRNA expression, and eventually promote target mRNA degradation or translational inhibition [18]. Several studies have reported miR-210, as one of the highly up-regulated miRNAs in hypoxic cells that is involved in numerous biological processes of the human body involving regulating mitochondrial metabolism, promoting the angiogenesis, proliferation and apoptosis. miR-210 has two versions, miR-210-3P and miR-210-5P. miR-210-3p is the guide-strand that integrates into the RISC (RNA induced silencing complex), whereas miR-210-5p is the passenger-strand that is inactivated through degradation [19, 20]. miR-210-5P is up-regulated in several malignant tumors that are associated with a variety of functionally important targets involved in cancers [21]. However, whether miR-210-5p could regulate mitophagy to participate in drug-sensitivity remains to be examined in depth.

Hepatocellular carcinoma (HCC) is the sixth most common fatal malignancy and the major cause of cancer-related deaths worldwide [22]. Most patients are diagnosed at the advanced stage which is highly recalcitrant to the current therapies [23]. The multi-target tyrosine kinase inhibitor sorafenib is the FDA-approved first-line systematic therapy for advanced HCC patients, which increased median survival from 7.9 to 10.7 months [24]. It blocks tumor cell proliferation and angiogenesis by inhibiting the Rad/Mitogen-activated protein (MAP)/extracellular signal-regulated kinase (ERK)/ MEK signaling cascade, as well as the kinase activity of vascular endothelial growth factor receptor (VEGFR) and platelet-derived growth factor receptor (PDGFR)-β. However, almost all HCC patients have developed sorafenib resistance within a few months [25]. Since the drug targets several signaling pathways, the tumors acquire resistance through different mechanisms, such as activation of compensatory signaling cascades and a hypoxic microenvironment [26]. It is crucial to understand the precise molecular mechanisms underlying sorafenib resistance in order to identify novel therapeutic targets and improve the clinical outcome of HCC patients. Although, sorafenib-induced autophagy contributes to drug resistance, it is unknown whether hyperactivated mitophagy is also involved in sorafenib resistance in HCC [27–29]. Regarding hypoxia is a major stimulus of mitophagy and also an important cause of sorafenib resistance [30–32], our objective is to determine the potential relationship between mitophagy and hypoxia-induced sorafenib resistance in HCC. We found that hypoxia induced sorafenib resistance in hepatoma cells was accompanied by hyperactivated mitophagy and a downregulation of ATAD3A expression. Thus, ATAD3A is a crucial mediator of hypoxia induced mitophagy signaling in HCCs and a novel therapeutic target for reversing sorafenib resistance.

## Materials and methods

### Patients and tissue microarray

 Liver cancer tissues and paried normal tissues were collected form the First Affiliated Hospital of Third Military Medical University and Shanghai Biochip Company Ltd (Shanghai, China). The informed consent was obtained from all patients. 

### Cell culture

Human hepatoma cell lines Huh7 and LM3 were obtained from the American Type Culture Collection (ATCC) and authenticated by the Cell Bank of Type Culture Collection of Chinese Academy of Science. The cells were maintained under recommended conditions. For hypoxia treatment, the cells were either cultured in a sealed hypoxia chamber (Thermo fisher, Inc.) containing 1% O_2_, 5% CO_2_ and 94% N_2_, or treated with 100 mM CoCl_2_ for 24 or 48 h. Sorafenib-resistant Huh7 (Huh7-SR) and LM3 (LM3-SR) cells were enriched by steadily increasing the drug dose, while hypoxia-induced sorafenib resistant cell lines (Huh7-H-SR and LM3-H-SR) were established by culturing under 1% O_2_ with increasing drug doses.

### Virus production

The pLVX-CMV-EGFP-3FLAG-PGK-Puro lentiviral vector expressing full-length human ATAD3A was purchased from SunBio (Shanghai. China). The pMAGic2.1-CMV-HygroR-U6 shRNA lentivirus vector purchased from SunBio (Shanghai. China), and the shRNA sequences are shown in Additional file 1. Human HEK293T cells (American Type Culture Collection) were cultured in 6-well plates till ~ 70% confluent, and co-transfected with 2 μg overexpression or knockdown virus vector, 1 μg pMD2.G and 1 μg psPAX2 lentivirus packaging vectors using Lipofectamine 2000 (Invitrogen) according to the manufacturer’s protocol. The cells were maintained in high-glucose DMEM containing10% FBS, 2 mM glutamine, and100 units/ml penicillin and streptomycin. The supernatants with virus were harvested twice at 48 h and 72 h, filtered through a 0.45 μm syringe filter and frozen in liquid nitrogen.

### Target cell transduction

HCC cells were cultured in complete DMEM, and 1.5 × 10^5^ cells were mixed with 450 μl virus-containing supernatant in the presence of 4 μg/ml polybrene (Sigma). The cells were seeded into a 6-well plate, and the medium was changed 12 h post-infection. After 48 h, the infected cells were trypsinized and seeded into 10 cm culture dish with 4 μg/ml puromycin (Thermo Fisher Scientific). The stably transduced cells were selected over 48 h, and harvested 6 days post-infection to determine knockdown efficiency.

### Western blotting

Total protein was extracted from cancer cells using Mammalian Protein Extraction Buffer (P0013, Beyotime, Beijing, China) supplemented with protease inhibitor cocktail (87786, ThermoFisher, USA). The mitochondrial fractions were separated using Mitochondria Isolation Kit (number: SM0020, Solarbio, Beijing, China) according to the manufacturer’s protocol, and the protein was extracted as above. Equal amounts of protein lysates were separated by SDS-PAGE gel and electro-transferred to PVDF membrane (Millipore, USA). After blocking in 5% milk-PBST for 2 h at 37 °C, the membranes were incubated overnight with primary antibodies (Additional file 2) at 4 °C and with secondary antibodies at 37 °C for 2 h. The positive bands were visualized with Immobilon Western Chemiluminescent HRP Substrate detection reagent (Millipore, USA), and acquired using a ChemiDoc™ imaging System (Bio-Rad, USA).

### Quantitative real-time PCR

Total RNA was extracted from cancer cells using Trizol Reagent (Invitrogen, USA), and qRT-PCR was performed using SYBR Prime Script RT-PCR kit (TaKaRa, Japan) on a Rotor-Gene 6000 real-time genetic analyzer (Corbett Life Science, USA). The primer sequences and the product sizes are listed in Additional file 3. Glyceraldehyde-3-phosphate dehydrogenase (GAPDH) was used as the internal control. The PCR conditions were as follows: denaturation at 95 °C for 2 mins, followed by 40 cycles of amplification and quantification (95 °C for 5 s, 55 °C–57 °C for 30 s), and melting curve (55 °C–95 °C, with 0.5 °C increment each cycle). Each sample was tested in triplicates.

### Apoptosis assay

For apoptosis assays, the suitably treated cells were washed twice with cold PBS, and resuspended in binding buffer at the density of 1 × 10^6^ cells/ml, and distributed into 500 μl aliquots (1 × 10^5^) in 2 ml tubes. 5 μL of Annexin V-FITC were added to each tube. Cells were incubated at room temperature (25 °C) for 15 min in a dark environment, and then analyzed on a FACS Calibur (BD Biosciences, USA), and results were calculated using Cell Quest software (BD Biosciences, USA).

### Immunofluorescence assay

HCC cells were washed, fixed with 5% paraformaldehyde (PFA) and permeabilized in 0.1% Triton X-100. After incubating overnight with monoclonal mouse anti-HIF-1α antibody (Cell signaling technology, #79233) or monoclonal rabbit anti-ATAD3A antibody (Invitrogen, PA5–03671) at 4 °C, the cells were probed with fluorescein isothiocyanate (FITC)-labeled anti-mouse IgG or Cy3-labeled anti-rabbit IgG secondary antibodies (Santa Cruz Biotechnology, Santa Cruz, USA). The nuclei were counterstained with Hochest 33258 and observed under laser confocal scanning microscopy (Leica TCS-SP5, Germany).

### Luciferase reporter assay

The 3′-UTR region of human ATAD3A was amplified by PCR from genomic DNA and cloned downstream to the firefly luciferase coding region in the pMIR-REPORTTM plasmid. The 293 T cells were seeded in 96-well plates, and co-transfected with 100 ng/ml reporter plasmid and 50 nM miR-210-5P or NC mimics using Lipofectamine 2000 24h late. Luciferase activity were measured after 72 h using the Dual-Glo Luciferase Assay System (Promega, Madison, WI, USA). All experiments were performed in triplicates.

### Cell proliferation assay

Cells were seeded in 96-well plates at the density of 5000 cells/well. After treating with 5.13 μM (Huh7) or 7.92 μM (LM3) sorafenib for varying durations (0 h,12 h,24 h,48 h and 72 h), the cells were incubated for 2 h with 0.5 mg/ml 3-(4, 5-dimethyl-thiazol-2-yl)-2,5-diphenyl-tetrazolium bromide (MTT) (Sigma-Aldrich) in serum free medium. To measure the IC50 of different cell lines, 10^− 3^,10^− 2^,10^− 1^,10^0^,10^1^ and 10^2^μM sorafenib were added in the 96-well plates for 72 h, then treated same as above. The optical densities were measured at 450 nM spectral wavelength using the microplate reader (ELx800; Bio-Tek Instruments, Inc.).

### Colony formation assay

For colony formation assay, 5 × 10^2^ cells were seeded in 6-well plates with sorafenib 5.13 μM (Huh7) or 7.92 μM (LM3) as appropriate. The conditioned medium was removed 3 days later and replaced by medium containing 10% serum for 2 weeks. The ensuing colonies were stained by crystal violet and counted. The experiment was performed thrice.

### Mitophagy assay

Western blotting was used to test LC3 mobility shift by analyzing the expression levels of LC3 I/II, and mitochondrial marker protein TOMM20 and TOMM70. Subcellular localization of mitophagy bodies was tracked by transmission-electron-microscopy (TEM, JEM-1230, Japan). The cells were fixed in cooling 2% glutaraldehyde and 0.1 M cacodylate buffer at 4 °C overnight, then exposed to phosphate buffer containing 1% osmium tetroxide for 1 h. Following dehydrated in different concentrations of acetone, these cells were infiltrated and embedded into Epon. The embedded cells were sectioned and stained with 3% uranyl acetate and lead citrate. Mitochondrial content was analyzed by Mito-Tracker staining (number: C1049, Beyotime Biotechnology, Shanghai, China) according to the manufacturer’s protocol and then analysed by FACS (BD Biosciences, USA), and results were calculated using Flow Jowo software (BD Biosciences, USA).

### Lactate measurement

The suitably treated cells were seeded in 6-well plates in triplicate at the density of 5*10^4^ cells/well. After culturing for 2 days, the number of viable cells was counted, and the culture medium was collected. Lactate levels were detected in the latter using a specific analytical kit (Nanjing Jian cheng Bioengineering Institute, China), and normalized to cell number and calculated as relative units per cell.

### Immunohistochemistry (IHC)

IHC was performed on tissue array slides as previously described. The tissues were probed with rabbit anti-human ATAD3A (Invitrogen, PA5–03671 1:200) and rabbit anti-human HIF-1α (abcam, ab51608, 1:200) antibodies, and counterstained with hematoxylin (Sigma). The staining intensity (negative = 0, weak = 1, moderate = 2, or strong = 3) and the percentage of positively stained cells (<5%=0, 5% to < 25% = 1, 25 to 50% = 2, > 50 to < 75% = 3, ≥75% = 4) were scored independently by two pathologists. The staining index was calculated by multiplying the intensity score with percentage score, and the samples were classified as negative/low expression or positive/high expression accordingly.

### RNA sequencing and bioinformatic data analysis

LM3-shControl and LM3-shATAD3A cells were generated as before and total RNA was extracted. RNA libraries were constructed using a TruSeq Stranded mRNA LT Sample Prep Kit (Illumina, San Diego, CA, USA) according to the manufacturer’s instructions, and sequenced on an Illumina platform (HiSeqTM 2500 or Illumina HiSeq X Ten) into 125 bp/150 bp paired-end reads by OE Biotech (Shanghai, China). The differentially expressed genes (DEGs) were identified using Cuff-diff with p value < 0.05 and fold change > 2 as the criteria. Gene set enrichment analysis was performed using software.broadinstitute.org/gsea. Heatmaps were generated with the heatmap package of R program.

### Tumorigenicity analysis in vivo

Female BALB/c (nu/nu) athymic nude mice, 5 weeks of age, were purchased from hfkbio Inc. Mice were maintained in specific pathogen-free conditions: 20–24 °C, 12/12 h of dark/light cycle, 60 ± 5% of humidity, and plastic cage (3–4 mice/cage). Bedding materials were changed every week, and environmental enrichment was done with sterile materials. All animal experiments were approved by the Institutional Animal Care and Use Committee of the Medicine University of Electronic Science and Technology of China’s. Control and miR-210-5P antagomir-transfected Huh7-shControl and Huh7-shATAD3A cells were inoculated subcutaneously (*n* = 5 each, 2 × 10^6^ cells per mouse) in the right flank of 4–6 week old BALB/c athymic female nude mice. Once the tumors grew to approximately 100mm^3^ (around 5 days), the mice were intraperitoneally injected with sorafenib tosylate (10 mg/kg) daily for a week, and monitored every 4 days for the appearance of subcutaneous tumors. We conducted the sacrifice of mice at 28 days, placed the mice in the chamber and introduced 100% carbon dioxide. After we removed each tumor, we maintained the carbon dioxide flow for a minimum of 1 min after respiration ceases. The tumor weight and tumor volume (TV; mm3) were calculated. Tumor volume was calculated as d2 × D/2 (d and D represent the shortest and the longest diameters, respectively). All animal experiments were performed in accordance with the guidelines of the ARRIVE reporting guidelines.

### Statistical analysis

Statistical analyses were performed using GraphPad Prism 6.0 (GraphPad, La Jolla, CA, USA). Two tailed unpaired t-test or one-way ANOVA were used to compare two or multiple groups. Survival curves were plotted using the Kaplan–Meier method and compared by the log-rank test. All data was presented as mean ± SD or mean ± SEM. *P* < 0.05 was considered statistically significant.

## Results

### Hyperactivated mitophagy plays a vital role in hypoxia-induced sorafenib resistance

**Fig. 1:**
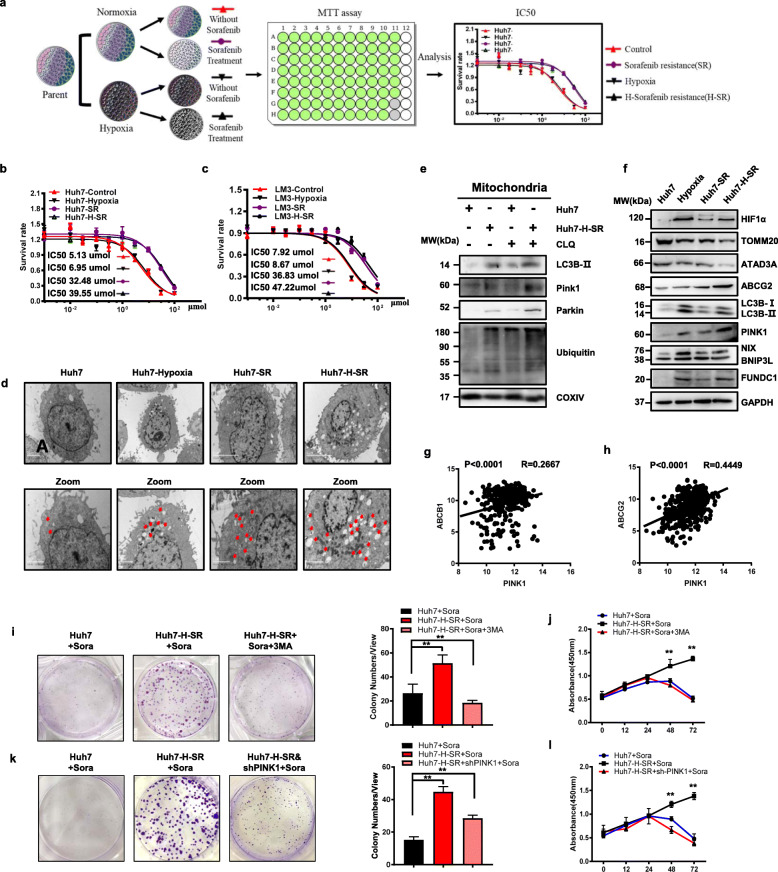
Hypoxia-induced sorafenib resistance in HCC cells is dependent on hyperactived mitophagy. **a** Schematic diagram showing establishment of sorafenib resistant (SR) and hypoxia-induced sorafenib resistant (H-SR) cells. **b, c** Evaluation of the half inhibitory concentration (IC50) of sorafenib in the indicated groups. **d** Transmission electron micrographs showing mitophagy in the indicated cells. **e** Immunoblots showing expression levels of the indicated proteins in control and Huh7-H-SR cells treated with/out chloroquine (CLQ). **f** Immunoblot showing expression levels of indicated proteins in control, hypoxia, SR and Huh7-H-SR cells. **g, h** Correlation between PINK1 expression and ABCB1 (**g**) and ABCG2 (**h**) in HCC patients from TCGA database. **i** Number of colonies formed by Huh7, Huh7-H-SR and Huh7-H-SR cells with 3MA (dosage: 5 mM) under sorafenib treatment (dosage: 5.13 μM). **j** Cell viability of Huh7, Huh7-H-SR and Huh7-H-SR cultured with 3MA under sorafenib treatment for varying durations. **k** Number of colonies formed by Huh7, Huh7-H-SR and Huh7-H-SR transfected with shPINK1 under sorafenib treatment. **l** Percentage of viable Huh7, Huh7-H-SR and Huh7-H-SR transfected with shPINK1 under sorafenib treatment for varying durations. Data are shown as mean ± SEM from three independent experiments, **p < 0.01, ***p < 0.001

As shown in Supplementary Fig. S1a-1d, HCC cells cultured under hypoxia showed significantly lower apoptosis rates and better cell viability response to sorafenib treatment in a time-dependent manner. To further analyze the role of hypoxia in sorafenib resistance, we established sorafenib resistant Huh7 (Huh7-SR) and LM3 (LM3-SR) cell lines, as well as the corresponding hypoxia-induced sorafenib resistant cell lines (Huh7-H-SR and LM3-H-SR) in the presence of increasing doses of sorafenib under 1% O_2_ culture condition (Fig. 1a-c). Compared to the respective control, Huh7-H-SR and LM3-H-SR cells showed increased levels of HIF-1α and drug resistance-related genes, including ABC20, ABCB1, ABCC1 and LRP (Supplementary Fig. S1e). Interestingly, extensive mitophagy was observed in Huh7-H-SR cells compared to the untreated controls, hypoxia treated Huh7 and Huh7-SR cells (Fig. 1d). Consistent with this, the Huh7-H-SR cells showed highest level of PINK1 expression, along with accumulation of PINK1, Parkin and LC3-II in the mitochondrial fraction (Fig. 1e). We also characterized a higher ubiquitination of multiple mitochondrial proteins via Parkin in Huh7-H-SR cells (Fig. 1e). Hypoxia-induced sorafenib resistant Huh7 and LM3 cells lines significantly upregulated PINK1, LC3-II, HIF-1α, FUNDC1 expression, and downregulated TOMM20 and ATAD3A expression (Fig. 1f). Furthermore, TCGA analysis results showed that PINK1 levels were positively correlated with that of ABCB1 and ABCG2 in HCC patients (Fig. 1g-h), and HIF-1α was positively correlated with ABCC1 and negatively with TOMM20 and TOMM70 (Supplementary Fig. S1f-1 h). These results indicated that activated mitophagy is the likely cause of hypoxia-induced sorafenib resistance. In agreement with this, the mitophagy inhibitor 3MA sensitized Huh7-H-SR cells to sorafenib, resulting in a significant decrease in colony yield and proliferation rates (Fig. 1i-j). Furthermore, PINK1 knockdown in Huh7-H-SR cells also sensitized the cells to sorafenib (Fig. 1k-l). Taken together, hyperactivated mitophagy plays a pro-survival role in the hypoxia-induced sorafenib resistant HCC cells.

### Hypoxia-induced sorafenib resistance is dependent on ATAD3A-mediated mitophagy

**Fig. 2:**
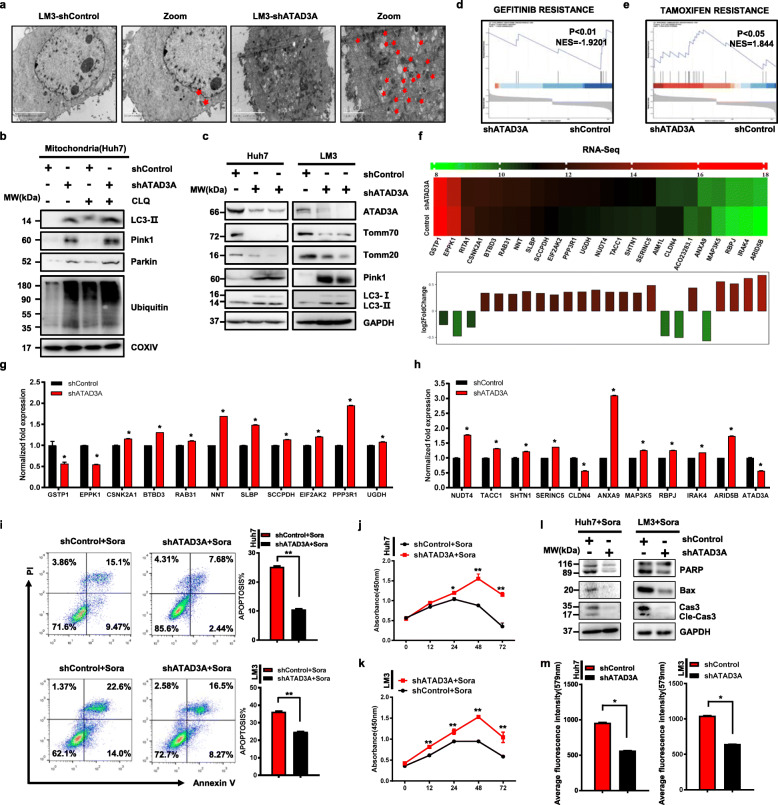
ATAD3A plays a vital role in sorafenib resistance and hyperactivates mitophagy in hepatoma cells. **a** Electron micrographs showing mitophagy in ATAD3A-knockdown LM3 cells. **b** Immunoblot showing expression levels of indicated proteins in untreated and chloroquine (CLQ)-treated Huh7 and Huh7-shATAD3A cells. **c** Immunoblot showing expression levels of indicated proteins in Huh7-shControl, Huh7-shATAD3A, LM3-shControl and LM3-shATAD3A cells. **d, e** GSEA of drug resistance genes in shControl and shATAD3A cells. NES, normalized enrichment score. **f** Heatmap showing expression for drug resistance core-enriched genes in LM3-shATAD3A cells relative to that in LM3-shControl cells. **g, h** qRT-PCR analyses of the indicated genes in shATAD3A and shControl LM3 cells. **i** Percentage of apoptotic cells in ATAD3A-knockdown cells. Annexin V and PI respectively indicate the early and late stage apoptotic cells. **j, k** Percentage of viable shControl and shATAD3A Huh7 (**j**) and LM3 (**k**) cells under sorafenib treatment for varying durations. **l** Immunoblot showing the expression levels of the indicated proteins in Huh7-shControl, Huh7-shATAD3A, LM3-shControl and LM3-shATAD3A cells. **m** Mito-Tracker fluorescence intensities in the indicated Huh7 cells and LM3 cells. Data are shown as mean ± SEM from three independent experiments, **p < 0.01, ***p < 0.001

The mitochondria transmembrane protein ATAD3A was significantly downregulated in the Huh7-H-SR and LM3-H-SR cells compared to the respective controls (Supplementary Fig. S2a). To determine the biological relevance of ATAD3A in HCC, we established stable ATAD3A-overexpressing and knockdown Huh7 and LM3 cell lines (Fig. 2c and Supplementary Fig.S2b-2c). Mitophagy was hyperactivated in the shATAD3A LM3 cells compared to the control (Fig. 2a), which paralleled a marked increase in the polyubiquitination and accumulation of global mitochondrial proteins as seen in the hypoxia-induced sorafenib resistant cells (Fig. 2b). In addition, PINK1 and LC3-II were significantly upregulated, and TOMM20, TOMM70 were downregulated following ATAD3A knockdown (Fig. 2c). Transcriptome sequencing analysis of the control and shATAD3A LM3 cells identified 319 upregulated and 302 downregulated genes in the latter (Supplementary Fig. S2i-2j). Gene set enrichment analysis (GSEA) (Fig. 2f) showed that the shATAD3A cells were commonly enriched with drug resistance genes (NES = -1.939, *P* = 0.00828 for GEFITINIB resistance; NES = 1.8435; *P* = 0.02 for TAMOXIFEN resistance) (Fig. 2d, e). The expression levels of these genes were validated by qRT-PCR (Fig. 2g, h). We also confirmed that the expression level of these genes in LM3-H-SR were similar to that in sh-ATAD3A cells compared to shControl (Supplementary Fig. S2f). In addition, shATAD3A and hypoxia-induced sorafenib resistantLM3 cells desensitized the HCC cells to sorafenib, resulting in a significant decrease in apoptotic index (Fig. 2i and Supplementary Fig. S2g-2 h), whereas ATAD3A overexpression augmented the sorafenib-induced cell death (Fig. 2j, k and Supplementary Fig. S2d, 2e). Loss of ATAD3A downregulated the pro-apoptotic PARP, Bax and Cleaved-Caspase 3 expression (Fig. 2l), decreased mitochondrial mass (Fig. 2m), and increased lactate production (Supplementary Fig. S3a-3b) in Huh7 and LM3 cells. Taken together, ATAD3A plays a vital role in mitophagy and sorafenib insensitivity in HCC cells.

### ATAD3A participates in hypoxia-induced mitophagy and sorafenib sensitivity

**Fig. 3:**
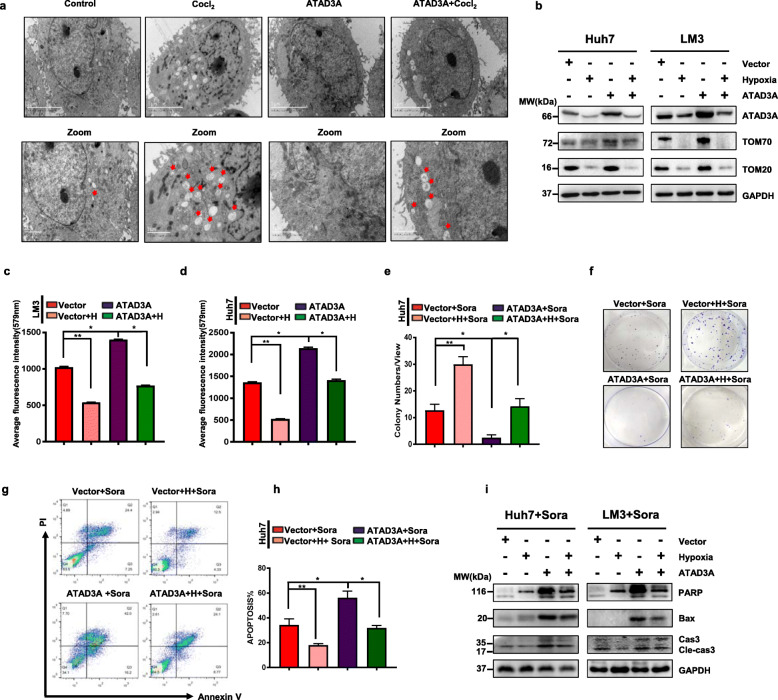
ATAD3A plays a vital role in hypoxia-induced mitophagy and sorafenib sensitivity in hepatoma cells. **a** Electron micrographs showing mitophagy in ATAD3A-overexpressing Huh7 cells treated with or without CoCl_2_. **b** Immunoblot showing the expression levels of indicated proteins in empty vector and Ov-ATAD3A cells with/out CoCl_2_ treatment. **c, d** Mito-Tracker fluorescence intensities of the indicated LM3 (**c**) and Huh7 (**d**) cells. **e, f** Number of colonies formed by vector and ATAD3A overexpression Huh7 cells under normal or hypoxia condition with sorafenib treatment. **g, h** Apoptosis rates in vector and ATAD3A overexpression LM3 cells under normal or hypoxia condition with sorafenib treatment. Annexin V and PI respectively indicate the early and late stage apoptotic cells. **i** Immunoblot showing expression levels of indicated proteins in Huh7-vector, Huh7-Ov-ATAD3A, LM3-vector and LM3-Ov-ATAD3A cells with/without CoCl_2_ under sorafenib treatment. Each bar represents the mean ± SEM of three independent experiments. *P < 0.05 and ** *P* < 0.01

To further determine the biological significance of hypoxia and ATAD3A expression in HCC cells, we introduced ectopic ATAD3A in the hypoxia cultured cells. As shown in Fig. 3a, hypoxia-induced mitophagy was abrogated by ATAD3A overexpression, which also upregulated TOMM20 and TOMM70 in Huh7 and LM3 cells (Fig. 3b). Ectopic ATAD3A also restored the mitochondrial mass (Fig. 3c, d) and decreased lactate concentration (Supplementary Fig. S3c, 3d) upon hypoxia-treated LM3 and Huh7 cells. Under hypoxia condition, cancer cells were insensitive to sorafenib treatment, while, gain of ATAD3A expression mitigated the insensitivity as indicated by the fewer colonies (Fig. 3e, f) and increased apoptotic index in LM3 cells (Fig. 3g, h). Western blot confirmed that PARP, Bax and Cleaved-Caspase3 were downregulated in hypoxia cells which were restored in ATAD3A overexpression cell under sorafenib treatment. Furthermore, we confirmed that ATAD3A expression restored sorafenib sensitivity in hypoxia treated Huh7 and LM3 cells via the apoptotic related proteins (Fig. 3i).

### ATAD3A is a direct functional target of hypoxia-induced mir-210-5p

**Fig. 4:**
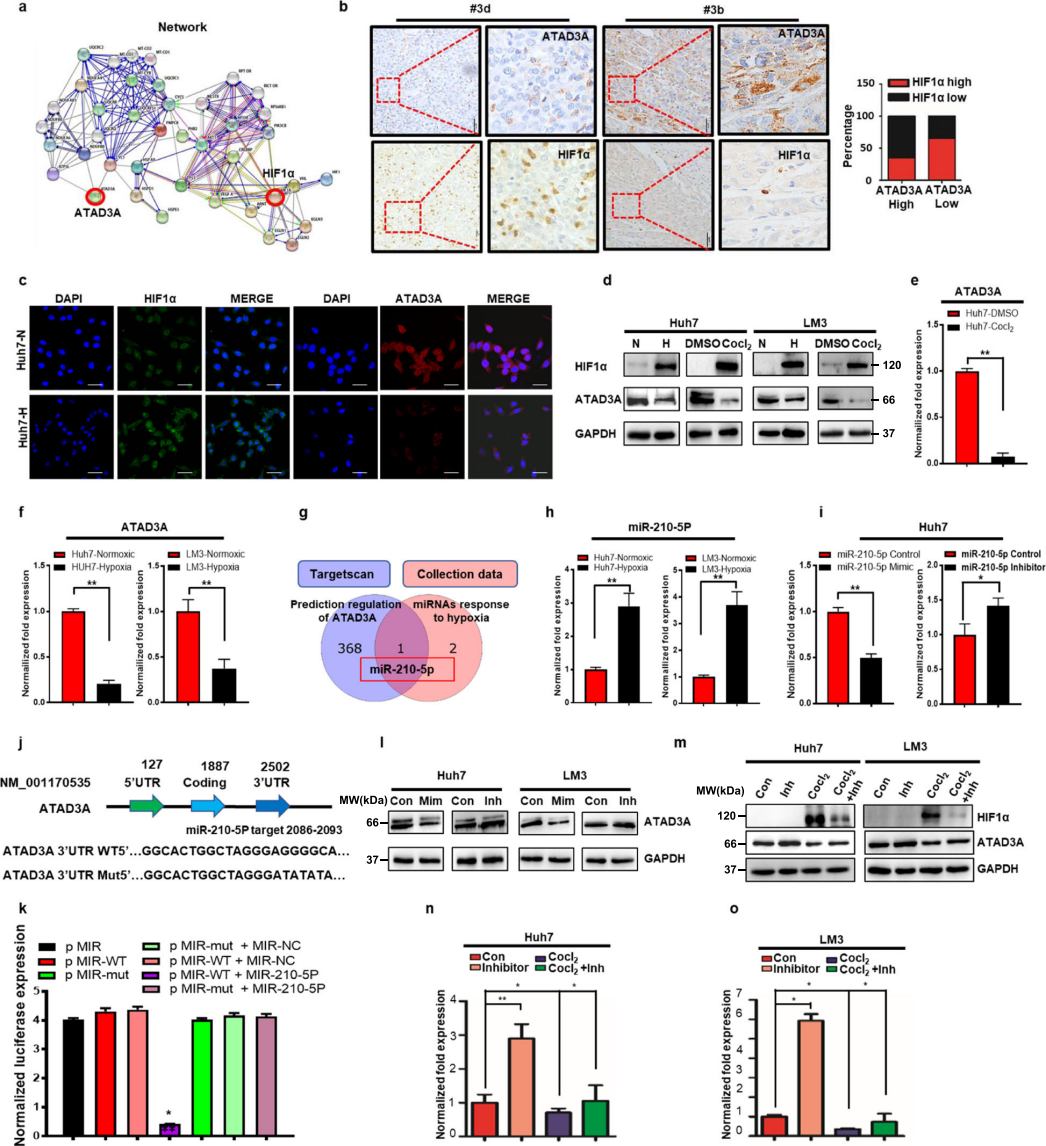
ATAD3A is a direct target of hypoxia-induced miR-210-5p in hepatoma cells. **a** Gene-gene interaction network. **b** In situ ATAD3A and HIF-1α expression in paired HCC and normal liver tissues (*n* = 85). **c** Representative immunofluorescence images showing HIF-1α (in green) and ATAD3A (in red) expression Huh7 cells cultured under normoxic and hypoxic (1% O_2_ for 24 h) conditions. Scale bar = 200 μm. **d** Immunoblot showing HIF-1α and ATAD3A in LM3 and Huh7 cells under 1% O_2_ or CoCl_2_ treatment for 24 h. **e** qRT-PCR analysis showing ATAD3A mRNA levels in Huh7 cells treated with CoCl_2_ or DMSO for 24 h. **f** qRT-PCR analysis showing ATAD3A mRNA levels in Huh7 and LM3 cells under 1% or 20% O_2_. **g** The overlap between the predicted miRNA regulators of ATAD3A and the hypoxia-responsive miRNAs from three different digestive tract cancers. **h** qRT-PCR analysis showing miR-210-5P levels in Huh7 and LM3 cells treated with 20% or 1% O_2_. **i** qRT-PCR analysis showing the expression of ATAD3A in Huh7 cells treated with miR-210-5P mimic or inhibitor. **J, k** The binding sites of miR-210-5p in the 3’UTR region of ATAD3A based on bioinformatics prediction and the sequences of designed ATAD3A mutants (**j**). Luciferase reporter assay of 293 T cell transfected with miR-210-5P mimics or miR-NC and ATAD3A-3’UTR-wt or ATAD3A-3’UTR-mut (**k**). **l** Immunoblot showing ATAD3A expression in hepatoma cells transfected with miR-210-5P mimic or inhibitor. **m-o** Immunoblot (**m**) and qRT-PCR (**n**-**o**) analyses of ATAD3A expression in Huh7 and LM3 cells transfected with miR-210-5P inhibitor with/without CoCl_2_ treatment. Each bar represents the mean ± SEM of three independent experiments. *P < 0.05 and ** *P* < 0.01

To determine the mechanism underlying hypoxia-mediated regulation of ATAD3A expression, we performed a systematic bioinformatics analysis of gene-gene interaction networks based on mutations, copy number alterations, mRNA expression profiles, and protein expression profiles using the STRING database (http://string-db.org/cgi/network). ATAD3A and the hypoxia-responsive transcriptional factor HIF-1α are located in a network containing 40 nodes (Fig. 4a), suggesting a potential link between HIF-1α signaling and ATAD3A. Furthermore, in situ HIF-1α protein expression in 85 HCC tissues was negatively correlated with that of ATAD3A (Fig. 4b). As shown in Fig. 4c and Supplementary Fig. S4a, hypoxia treatment significantly down regulated ATAD3A expression in hepatoma cells. In addition, both 1% O_2_ and CoCl_2_ induced HIF-1α expression and downregulated ATAD3A by western blot in Huh7 and LM3 cells (Fig. 4d), and decreased ATAD3A expression in Huh7 cells as a time-dependent manner (Supplementary Fig. S4b). In addition, we found a loss of ATAD3A gene expression in 1% O_2_ and CoCl_2_ treated Huh7 and LM3 cells (Fig. 4e-f and Supplementary Fig. S4c). Bioinformatics analysis identified hsa-miR-210 and hsa-miR-193b as the most hypoxia-responsive miRNAs (Supplementary Fig. S4d), and ATAD3A as the potential target gene of miR-210-5P (Fig. 4g). Consistent with this, 1% O_2_ and CoCl_2_ increased miR-210-5p expression after 24 h (Fig. 4h and Supplementary Fig. S4e). Furthermore, miR-210-5P suppressed the expression of reporter gene carrying the wild-type but not mutant 3′-UTR of human ATAD3A gene (Fig. 4j, k). Hepatoma cells transfected with miR-210-5P mimic significantly inhibited ATAD3A expression, whereas miR-210-5p inhibitor had the opposite effect (Fig. 4i, l and Supplementary Fig. S4f). These findings indicated that ATAD3A is a direct target of the hypoxia induced miR-210-5P. This was confirmed by treating hepatoma cells with CoCl_2_ and/or the miR-210-5P inhibitor, which showed that hypoxia-induced downregulation of ATAD3A was abrogated by blocking miR-210-5P (Fig. 4m-o). Taken together, inhibition of ATAD3A under hypoxia is dependent on miR-210-5P.

### Hypoxia related sorafenib sensitivity and mitophagy are partially mediated by miR-210-5P

**Fig. 5:**
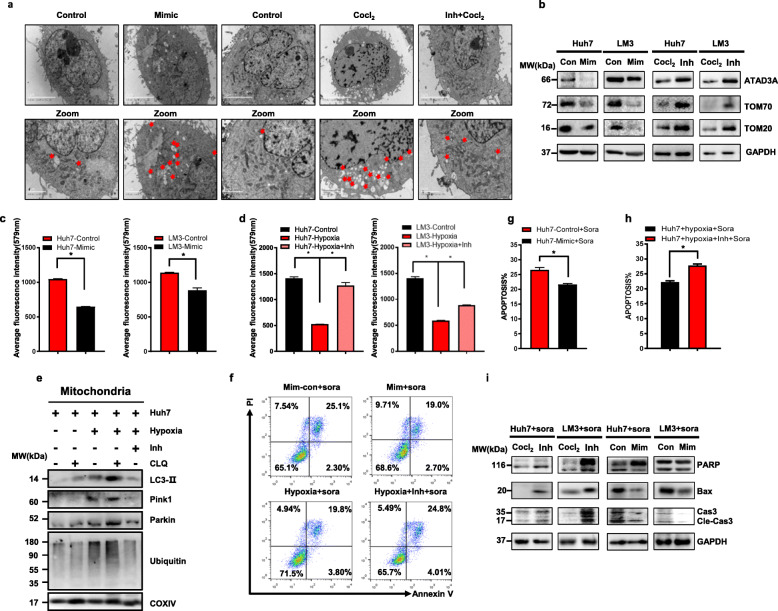
Hypoxia-induced sorafenib resistance and mitophagy are partially mediated by miR-210-5P. **a** Electron micrographs showing mitophagy in hypoxia-cultured Huh7 cells transfected with miR-210-5P mimic or inhibitor treated cells with or without CoCl_2_. **b** Immunoblot showing expression levels of indicated proteins in miR-210-5P mimic or inhibitor treated cells with or without CoCl_2_. **c, d** Mito-Tracker fluorescence intensities of the indicated huh7 (**c**) and LM3 (**d**) cells. **e** Immunoblot showing expression levels of indicated proteins in Huh7 cells treated with miR-210-5P inhibitor and 1% O_2_ with/without chloroquine. **f-h** Representative FACS analyses of apoptosis rates in Huh7 cells transfected with miR-210-5P mimic or inhibitor with or without hypoxia condition under sorafenib treatment. **i** Immunoblot showing expression levels of indicated proteins in miR-210-5P mimic or CoCl_2_ and miR-210-5P inhibitor-treated Huh7 and LM3 cells under sorafenib treatment. Each bar represents the mean ± SEM of three independent experiments. *P < 0.05 and ** *P* < 0.01

MiR-210 is a classic hypoxia-induced miRNA that can be activated by HIF-1α. Here, we found miR-210-5P mimic induced mitophagy in HCC cells that were similar to CoCl_2_ treatment. The hyper-activated mitophagy induced by CoCl_2_ could be reversed by miR-210-5P inhibitor transfection (Fig. 5a). Furthermore, miR-210-5P mimic downregulated TOMM20 and TOMM70 expression (Fig. 5b), decreased mitochondrial mass by flow cytometry (Fig. 5c), upregulated PINK1 expression level (Fig. 5e), and increased lactate production (Supplementary Fig. S3e-h) in the Huh7 and LM3 cells. Nevertheless, these could be neutralized by the miR-210-5P inhibitor in CoCl_2_-treated cells. As shown in Fig. 5d-h, depletion of miR-210-5P significantly increased the percentage of apoptotic cells in the presence of sorafenib, while the miR-210-5P mimic decreased apoptosis in the sorafenib-treated cells. The expression levels of the pro-apoptotic proteins differed accordingly in the miR-210-5P mimic/inhibitor-transfected cells (Fig. 5i). In conclusion, hypoxia-induced mitophagy is partially mediated by miR-210-5P.

### Clinical significance of ATAD3A expression in HCC and the miR-210-5P-ATAD3A axis in exnografts formation in vivo

**Fig. 6:**
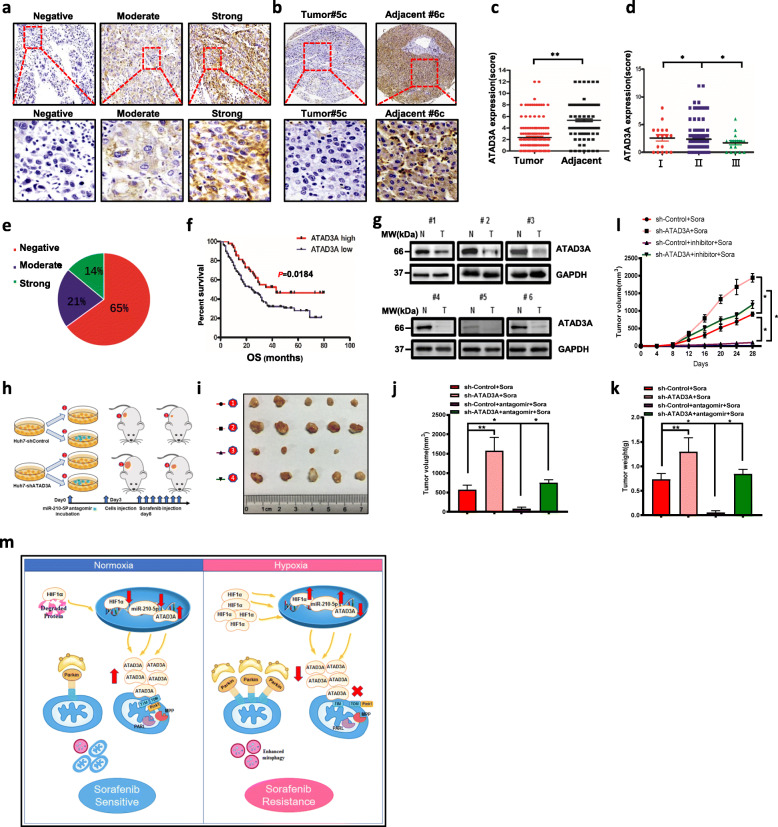
The miR-210-5P-ATAD3A axis influences xenografts formation in vivo. **a** Representative images of ATAD3A expression with IHC staining in hepatocellular carcinoma specimens. **b** Representative images of ATAD3A expression with IHC staining in tumor and adjacent hepatocellular carcinoma specimens. **c** Statistical analysis of IHC-determined ATAD3A expression in tumor and adjacent normal tissue from HCC patients (Student *t* test) (*n* = 85). **d** Comparison of IHC-determined ATAD3A expression among HCC patients of different pathological grades. **e** Percentages of strong, moderate, and negative ATAD3A expression in hepatocellular carcinoma samples(*n* = 135). **f** Kaplan-Meier analysis of ATAD3A expression and overall survival of HCC patients(*n* = 135). **g** Immunoblot analysis of ATAD3A expression in HCC tissues and their adjacent normal tissues. GAPDH was used as a loading control. **h**. Xenograft tumors derived from shATAD3A LM3 cells with/without miR-210-5P antagomir under sorafenib treatment. **i**-**l**. Representative images of tumor growth (**i**), and the tumor volume (**j**), tumor weight (**k**) and tumor growth curve (**l**) in the indicated groups. **m**. Schematic representation of ATAD3A-mediated mitophagy during hypoxia-induced sorafenib resistance in HCC cells. Each bar represents the mean ± SEM of three independent experiments. *P < 0.05 and ** *P* < 0.01

To investigate the expression level of ATAD3A in HCC tissues and their paired normal tissues. We generated a tumor sample tissue array containing 135 hepatocellular carcinoma samples with survival information and 85 cases with paired normal tissues to determine ATAD3A expression by IHC. Results showed that ATAD3A expression in HCC patients was heterogeneous, ranging from completely absent to strongly expressed and the representative images of hepatocellular carcinoma samples with strong, moderate or low ATAD3A expression were shown in Fig. 6a. ATAD3A expression level was lower in cancerous liver tissues compared to the adjacent normal tissues (Fig. 6b-c), and lower expression of ATAD3A was associated with higher-grade HCC patients (Fig. 6d). 14% of hepatocellular carcinoma samples had strong ATAD3A expression, whereas percentages of moderate and no ATAD3A expression were 21% and 65%, respectively (Fig. 6e). Kaplan–Meier analysis showed that lower ATAD3A expression in HCC was correlated closely with poor overall survival (OS) of the patients (*P* = 0.0184; Fig. 6f). Western blot analysis also showed that ATAD3A had a significantly lower expression in 6 HCC tissues compared to their paired normal tissues (Fig. 6g). Taken together, these data indicated that ATAD3A is down regulated in HCCs and indicative of poor clinical outcome of HCC patients. To further determine the tumorigenicity of sh-ATAD3A and whether miR-210-5P antagomir could attenuate the tumor progression role of sh-ATAD3A under sorafenib treatment in vivo. Huh7-shcontrol and Huh7-shATAD3A cells that have been transfected with or without antagomir of miR-210-5p construct were subcutaneously implanted into the right flank of nude mice (Fig. 6h). Results showed that sh-ATAD3A in Huh7 cells significantly increased the tumor weight and tumor volume compared to the control group in the presence of sorafenib treatment, and miR-210-5P antagomir reduced the tumor size and tumor volume both in control and sh-ATAD3A groups (Fig. 6i-l). Collectively, these data provide evidence for an important inverse regulating relationship between ATAD3A and miR-210-5P in vitro and in vivo. Taken together, our findings point to an inverse regulatory relationship between ATAD3A and miR-210-5P in mediating mitophagy in the sorafenib-resistant HCC cells under hypoxia (Fig. 6m).

## Discussion

Hypoxia is a hallmark of solid tumors and is the consequence of absent or abnormal vasculature in the tumor microenvironment. It is essential for tumor progression, angiogenesis, metastasis, invasion, immune escape and chemoresistance [33]. Various studies showed that sorafenib could stabilize HIF-1α expression under hypoxia and increase the expression of HIF-1α targets like MDR1, GLUT-1, VEGF and miRNAs (e.g. miR-210), and the whole progress played vital roles in cancer biology [34–36]. In addition, evidence indicated that hypoxia-induced mitophagy mainly depends on BNIP3/NIX and the FUNDC1 pathway [37, 38]. In the present study, we found that hypoxia induced mitophagy also dependent on the loss of ATAD3A, which could increase PINK1 accumulation in the outer mitochondrial membrane.

ATAD3A located on the mitochondrial membrane which regulated fragmentation, fission, protein transport and other aspects of mitochondrial physiology [39–42]. ATAD3A deletion triggered mitophagy through the PINK1/Parkin pathway in the hematopoietic system and induced cisplatin sensitivity by increasing mitochondrial fragmentation. In our present study, hypoxia-induced sorafenib resistance in HCC cells was associated with lower ATAD3A expression and upregulation of BNIP3/NIX, and loss of ATAD3A increased sorafenib insensitivity. Since PINK1 deletion nearly completely abrogated sorafenib resistance of Huh7 cells under hypoxia, we hypothesized that ATAD3A played a vital role in hypoxia-induced sorafenib resistance through the mitophagic pathway. Under both basal conditions and times of abnormal stress, the process of mitophagy was to prevent the accumulation of dysfunctional mitochondria and preserved mitochondrial homeostasis [43]. While, how mitophagy affected drug sensitivity was still confusing. The removal of damaged mitochondria through mitophagy could dampen the toxicity of chemotherapy [44]. Mitophagy is also described as a possible mechanism for preventing ROS production [45]. And cancer cells were likely to be more sensitive to the additional oxidative damage promoted by ROS [46–48]. Therefore, we presumed that hypoxia induced hyperactivated mitophagy may decrease ROS production and then result in sorafenib insensitivity.

HIF-1α is a key transcriptional factor that allows cells and tissues to adapt to hypoxia. Bioinformatics analysis predicted ATAD3A as a direct target of miR-210-5P, the predominant miRNA induced under hypoxia in multiple solid tumors and a surrogate marker for tumor hypoxia [49]. It promoted cancer cell proliferation, metabolism, angiogenesis, metastasis, invasion, chemo-resistance, and correlated with patient prognosis [50–53]. Mitochondrial homeostasis was crucial for cellular survival during hypoxia [52], and in line with this, miR-210-5P blockade reversed hypoxia-induced down-regulation of ATAD3A and sensitized the HCC cells to sorafenib by promoting mitophagy.

## Conclusions

In summary, the lack of reliable and robust predictive biomarkers of sorafenib sensitivity and treatment response has hindered the development of personalized therapy in HCC. Loss of ATAD3A increased HCC tumor growth in the presence of sorafenib treatment. However, miR-210-5P antagomir abolished the tumorigenic effect of ATAD3A knockdown. Thus, ATAD3A is an effective target for overcoming resistance to sorafenib and also a predictive marker for sorafenib response and clinical outcomes. Our findings also point to the role of ATAD3A-PINK1-Parkin signaling pathway in hypoxia-induced sorafenib resistance in HCC. Further investigation is warranted to examine the feasibility of miR-210-5P inhibition or ectopic ATAD3A expression combined with sorafenib as a novel therapeutic strategy against HCC. In addition, additional response predictors need to be assessed in order to identify patient subgroups most likely to benefit from sorafenib, achieve optimal risk-to-benefit ratio and maximize the treatment efficacy for each patient.

## Supplementary Information

**Additional file 1.** Target sequences and ORF expression clone for PINK1 and ATAD3A in the study.

**Additional file 2.** Antibodies used for immunostaining analysis in our dataset

**Additional file 3.** Sequences of the primers used for qRT-PCR.

**Additional file 4:** **Supplementary Fig. 1.** Hypoxia induced sorafenib resistance in hepatoma cells. a,b Sorafenib sensitivity under normoxic or hypoxia condition of LM3 and Huh7 cells measured by apoptosis rates (%). c, d Cell viability of LM3(c) or Huh7 (d) cells under hypoxic or normoxic conditions with sorafenib treatment. e qRT-PCR analysis of indicated genes in control, hypoxia treated, sorafenib resistant and hypoxia-induced sorafenib resistant Huh7 cells. f. Immunoblot showing expression levels of indicated proteins in control, hypoxia, SR and Huh7-H-SR cells. g-i Relation between HIF-1α expression and that of ABCC1 (g), TOMM20 (h) and TOMM70 (i) in HCC patients from TCGA database. Each bar represents the mean ± SEM of three independent experiments. *P < 0.05 and ** *P* < 0.01.

**Additional file 5:** **Supplementary Fig. 2.** ATAD3A plays a vital role in sorafenib sensitivity of hepatoma cells. a Immunoblot showing expression levels of indicated proteins in control and hypoxia-induced sorafenib resistant Huh7 and LM3 cells. b qRT-PCR analysis of ATAD3A expression in LM3-vector and ATAD3A overexpression cells. c Immunoblot showing expression levels of the indicated proteins in ATAD3A overexpressing Huh7 and LM3 cells. d, e Cell viability of Huh7-vector and Huh7-ATAD3A (e) or LM3-vector and LM3-ATAD3A (d) cells under sorafenib treatment. f qRT-PCR analyses of the indicated genes in LM3-H-SR, LM3-shATAD3A and LM3-shControl. g,h Percentage of apoptotic cells in LM3-H-SR, LM3-shATAD3A and LM3-shControl cells under sorafenib treatment. Annexin V and PI respectively indicate the early and late stage apoptotic cells. i DAVID analysis of the top 10 altered pathways using KEGG in shATAD3A LM3 cells compared to control. j The volcano plots showing the differently expressed genes in shATAD3A versus shcontrol cells. Each bar represents the mean ± SEM of three independent experiments. *P < 0.05 and ** *P* < 0.01.

**Additional file 6:** **Supplementary Fig. 3.** ATAD3A plays a vital role in lactate production of hepatoma cells. a,b Lactate levels in the supernatants of Huh7-shControl and Huh7-shATAD3A (a) or LM3- shControl and LM3-shATAD3A (b) cells. c,d Lactate levels in Huh7-Vector and Huh7-ATAD3A (c) or LM3-Vector and LM3-ATAD3A (d) cells with/without 1% O_2_ exposure for 24 h. e,f Lactate levels in Huh7 (e) and LM3 (f) cells with/ without miR-210-5P inhibitor transfection and with/ without CoCl_2_ treatment. g, h Lactate levels in control and miR-210-5P mimic-transfected Huh7 (g) and LM3 (h) cells. Each bar represents the mean ± SEM of three independent experiments. *P < 0.05 and ** P < 0.01.

**Additional file 7: Supplementary Fig. 4.** ATAD3A is down-regulated in hypoxia and a potential target of miR-210-5P. a Immunofluorescent staining of HIF-1α (green) and ATAD3A (red) expression in normoxic and hypoxic LM3 cells (1% O_2_ treatment for 24 h). Scale bar = 200 μm. b Immunoblot showing expression levels of indicated proteins in Huh7 cells with/without CoCl_2_ treatment for different durations. c qRT-PCR analysis of ATAD3A expression in LM3 cells with/without CoCl_2_ treatment. d Bioinformatics analysis of the hypoxia-responsive miRNAs from digestive cancers. e qRT-PCR analysis showing miR-210-5P levels in Huh7 and LM3 cells treated with DMSO or CoCl_2_ for 24 h. f qRT-PCR analysis showing the expression of ATAD3A in LM3 cells treated with miR-210-5P mimic and inhibitor. Each bar represents the mean ± SEM of three independent experiments. *P < 0.05 and ** P < 0.01.

## Data Availability

All data and materials generated or analysed during the current study are available from the corresponding author on reasonable request.

## Abbreviations

MIR: Myocardial ischemia reperfusion injury
AS: Ankylosing spondylitis
CRC: Colorectal cancer
PINK1: PTEN–induced putative kinase protein 1
HCC: Hepatocellular carcinoma
BNIP3L/NIX: Bcl-2/adenovirus E1B 19-kDa interacting protein 3 like
FUNDC1: FUN14 domain-containing protein 1
OS: Overall survival
HIF-1α: Hypoxia-inducible factor 1α
TOMM20: Mitochondrial import receptor subunit TOM20 homolog
TOMM70: Mitochondrial import receptor subunit TOM70 homolog
LC3: Microtubule-associated protein1light chain3
ABC20: ABC-type transport system permease protein
ABCB1: ATP-binding cassette subfamily B member 1
ABCC1: Multidrug resistance-associated protein 1
LRP: Lipoprotein Receptor-Related Protein
MRP1: Multidrug resistance-associated protein 1
IHC: Immunohistochemistry
qRT-PCR: Quantitative real-time PCR
