# Supplementary material for: Mitophagy promotes sorafenib resistance through hypoxia-inducible ATAD3A dependent Axis
Source: J Exp Clin Cancer Res. 2020 Dec 7;39:274. doi: 10.1186/s13046-020-01768-8 (PMC7720487; doi:10.1186/s13046-020-01768-8)
Supplement: Supplementary file 1 — Additional file 1. Target sequences and ORF expression clone for PINK1 and ATAD3A in the study. [file 13046_2020_1768_MOESM1_ESM.docx]

**Additional file 1. Target sequences and ORF expression clone for PINK1 and ATAD3A.**

| Gene  (Accession no.) | | Primer sequence (5’→3’) |
| --- | --- | --- |
| PINK1  (NM_65018)  ATAD3A  (NM_001170535 ) | Target sequence | 1- CCAGGCTGGGCCGCAGGACCG  2- CCCCTCACCCCAACATCATCC  3- CGGACGCTGTTCCTCGTTATG |
|  | Target sequence | CCGGCCTGCACATTTAGGATATGCTCTC  GAGAGCATATCCTAAATGTGCAGGTTTTTTG |
| ATAD3A  (NM_001170535 ) | ORF clone | Forward: 5'CTCCATAGAAGACACCGAC-3'  Reverse: 5-'CATATAGACAAACGCACAC3' |
