# Supplementary material for: Mitophagy promotes sorafenib resistance through hypoxia-inducible ATAD3A dependent Axis
Source: J Exp Clin Cancer Res. 2020 Dec 7;39:274. doi: 10.1186/s13046-020-01768-8 (PMC7720487; doi:10.1186/s13046-020-01768-8)
Supplement: Supplementary file 2 — Additional file 2. Antibodies used for immunostaining analysis in our dataset [file 13046_2020_1768_MOESM2_ESM.docx]

**Supplementary table2. Antibodies used for immunostaining analysis.**

| Antibody  (Accession no.) | Common name | Sources | Corp. |
| --- | --- | --- | --- |
| PA5-03671 | ATAD3A | Rabbit | Invitrogen |
| #42406S | TOMM20 | Rabbit | Cell Signaling |
| sc-390545 | TOMM70 | Mouse | Santa Cruz |
| #4477S | ABCG2 | Rabbit | Cell Signaling |
| #2118 | GAPDH | Rabbit | Cell Signaling |
| Ab92498 | HIF1α | Rabbit | Abcam |
| #79233 | HIF1α | Mouse | Cell Signaling |
| #4108S | LC3I/II | Rabbit | Cell Signaling |
| #6946 | PINK1 | Rabbit | Cell Signaling |
| Ab224722 | FUNDC1 | Rabbit | Abcam |
| #12396 | BNIP3L/NIX | Rabbit | Cell Signaling |
| #9532S | PARP | Rabbit | Cell Signaling |
| #2772 | BAX | Rabbit | Cell Signaling |
| #9662S | Caspase3 | Rabbit | Cell Signaling |
| #2132S | Parkin | Rabbit | Cell Signaling |
| #3933S | ubiquitin | Rabbit | Cell Signaling |
| #4844S | COX IV | Rabbit | Cell Signaling |
