# Supplementary material for: Mitophagy promotes sorafenib resistance through hypoxia-inducible ATAD3A dependent Axis
Source: J Exp Clin Cancer Res. 2020 Dec 7;39:274. doi: 10.1186/s13046-020-01768-8 (PMC7720487; doi:10.1186/s13046-020-01768-8)
Supplement: Supplementary file 3 — Additional file 3. Sequences of the primers used for qRT-PCR. [file 13046_2020_1768_MOESM3_ESM.docx]

**Additional file 3. Sequences of the primers used for qRT-PCR.**

| Gene  (Accession no.) | Primer sequence (5’→3’) | Amplification  size (bp) |
| --- | --- | --- |
| ATAD3A | F: GCGAGCCACCGAGAAGATAAG | 159 |
| ([NM_](http://www.ncbi.nlm.nih.gov/entrez/viewer.fcgi?db=nucleotide&id=83641890)001170536) | R: TGGACCATCTCATTGATGCGG |  |
| GAPDH  ([NM_002046](http://www.ncbi.nlm.nih.gov/entrez/viewer.fcgi?db=nucleotide&id=83641890)) | F: CAAGCTCATTTCCTGGTATGAC  R: CAGTGAGGGTCTCTCTCTTCCT | 142 |
| ABC20  (NM_000927) | F: AGGGAAAGTGCTGCTTGA  R: GCTGTTGTCTCCATAGGCA | 139 |
| ABCB1  (NM_000927) | F: CCGCTGTTCGTTTCCTT  R: TCTTTGCTCCTCCATTGC | 134 |
| HIF-1α  (NM_001243084) | F: CCCATTCCTCACCCATC | 127 |
|  | R: CTGGCTCATATCCCATCAA |  |
| LRP  (NM_002332) | F: GGGGACAAGCTGTGGTG  R: CCAGGGTGGTGCTGTTC | 103 |
| GSTP1 | F: AATACCATCCTGCGTCACC | 150 |
| (NM_000852) | R: CTTGCCCGCCTCATAGTT |  |
| EPPK1 | F: AGGGGCGCACGAGCTAT | 100 |
| (NM_031308) | R: GGGAGGGCAGGAGCACT |  |
| RITA1 | F: TCCGTGCTCTCTTGTGGA | 112 |
| (NM_001286215) | R: GGCTCTGTCTTGGAGGGA |  |
| CSNK2A1 | F: AGTGCCAACCCCTTCAC | 135 |
| (NM_001362770) | R: GCTCAGGCATCAGGAGAC |  |
| BTBD3 | F: GCAGATCGAGCCAGACA | 129 |
| (NM_001282550) | R: GAGGAGCACTGAAACTGGA |  |
| RAB31 | F: CTCAAAGTGTGCCTTCTCG | 141 |
| (NM_006868) | R: CCCCAGTGTCCTCCATC |  |
| NNT | F: TTGACTTTGGTACGATGGG | 113 |
| (NM_001331026) | R: TTTTACTGGGGCACCTTG |  |
| SLBP | F: AGTCCCAAGACACCTTCG | 117 |
| (NM_001306074) | R: AAATGCAGAGCCACCTTC |  |
| SCCPDH | F: GTAACTGTGGGAGGCATCA | 127 |
| (NM_016002) | R: AGCCAAAGGAGAAGAACCA |  |
| EIF2AK2 | F: GGCGCAGTTTGCTCATA | 127 |
| (NM_001135651) | R: GATGGACCTCGATGCCT |  |
| PPP3R1 | F: TCCCTTCCCATTTCTGC | 134 |
| (NM_000945) | R: TCAACCTGCCCTTTTCC |  |
| UGDH | F: AGAGGGAACAGCCATCAA | 121 |
| (NM_001184700) | R: GGGAACCCAGTGCTCATA |  |
| TACC1 | F: TGGCATCAGTAAGTCAGCA | 139 |
| (NM_001122824) | R: AACAGAGTGGCTCCCAAA |  |
| SHTN1 | F: CGCTCCCTCTTTCCCTT | 110 |
| (NM_001127211) | R: TTCGCCTATTGCTTGCTC |  |
| SERINC5 | F: CCCAACCACGTCTTTACTG | 158 |
| (NM_001174071) | R: CCCACTCTATCTCCCCTTC |  |
| CLDN4 | F: TCCTCTGTTCCGGGTAGG | 123 |
| (NM_001305) | R: CCATCCACTCTGCACTTCC |  |
| ANXA9 | F: TACAGGCAGCACTTTCCG | 123 |
| (NM_003568) | R: CCACGTCCACAGCAGAAT |  |
| MAP3K5 | F: CCTGACGGAAGACCAAGA | 105 |
| (NM_005923) | R: GCAATGAGGGTTGTGATGT |  |
| RBPJ | F: AGCAAGCGGATAAAAGTCAT | 104 |
| (NM_001080927) | R: CGATTAAACAGAGCCACCTT |  |
| IRAK4 | F: TTTGCCTTCCATTGTGATT | 103 |
| (NM_001114182) | R: TTGTTTTGGCTTACGGTTC |  |
| ARID5B | F: CCGGCAACTTTTATCCAG | 117 |
| (NM_001244638) | R: TTCACAATCACCTTTTCGG |  |
