# Supplementary figures and images for: Mitophagy promotes sorafenib resistance through hypoxia-inducible ATAD3A dependent Axis
Source: J Exp Clin Cancer Res. 2020 Dec 7;39:274. doi: 10.1186/s13046-020-01768-8 (PMC7720487; doi:10.1186/s13046-020-01768-8)

a

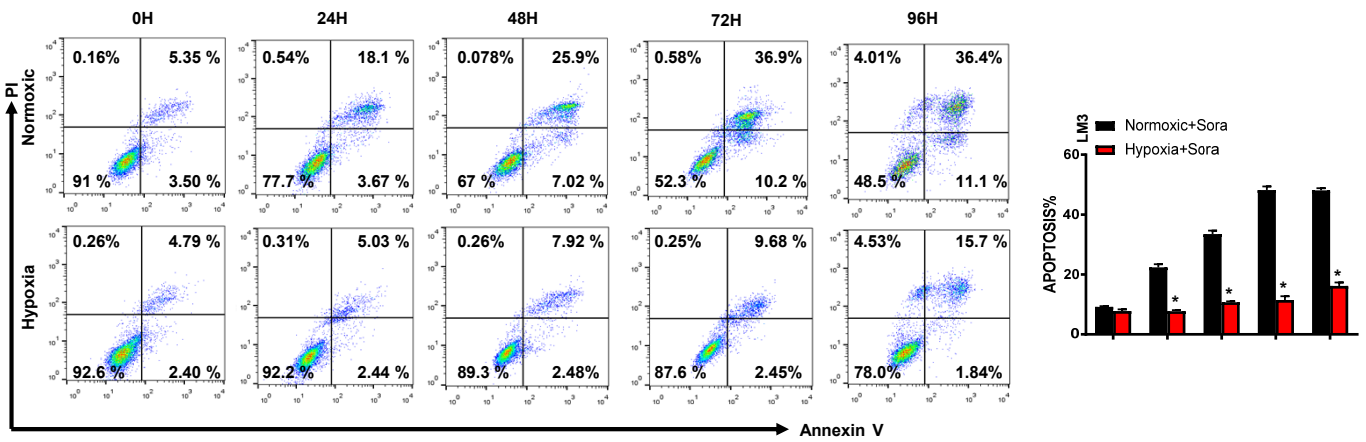

b

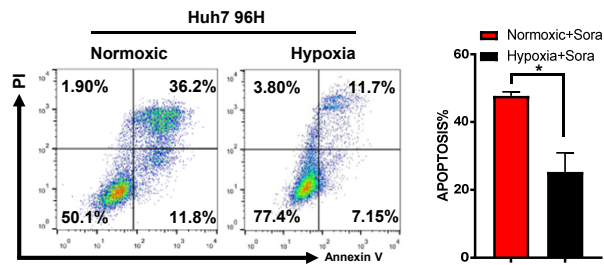

e

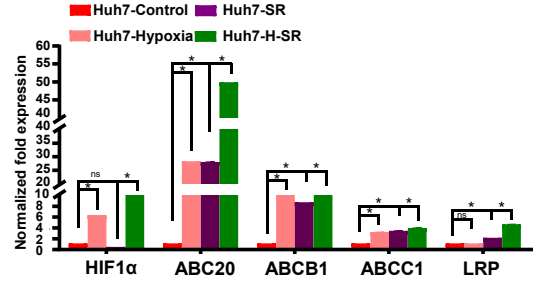

c

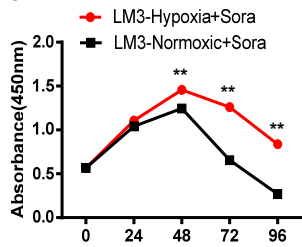

f

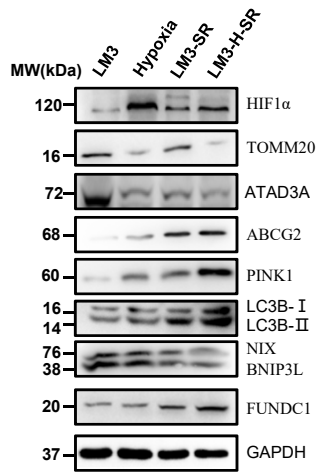

g

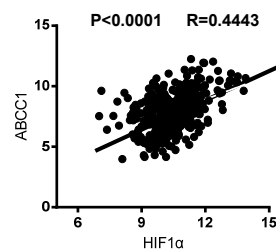

h

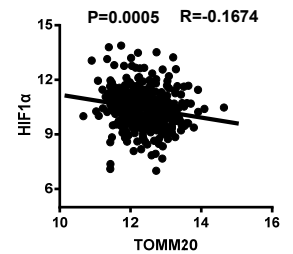

d

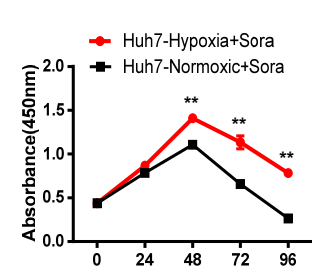

i

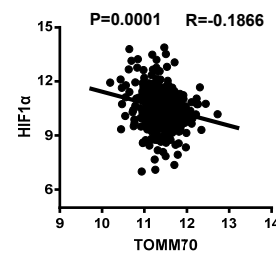

Supplement: Supplementary file 4 — Additional file 4: Supplementary Fig. 1. Hypoxia induced sorafenib resistance in hepatoma cells. a,b Sorafenib sensitivity under normoxic or hypoxia condition of LM3 and Huh7 cells measured by apoptosis rates (%). c, d Cell viability of LM3(c) or Huh7 (d) cells under hypoxic or normoxic conditions with sorafenib treatment. e qRT-PCR analysis of indicated genes in control, hypoxia treated, sorafenib resistant and hypoxia-induced sorafenib resistant Huh7 cells. f. Immunoblot showing expression levels of indicated proteins in control, hypoxia, SR and Huh7-H-SR cells. g-i Relation between HIF-1α expression and that of ABCC1 (g), TOMM20 (h) and TOMM70 (i) in HCC patients from TCGA database. Each bar represents the mean ± SEM of three independent experiments. *P < 0.05 and ** P < 0.01. [file 13046_2020_1768_MOESM4_ESM.pdf]

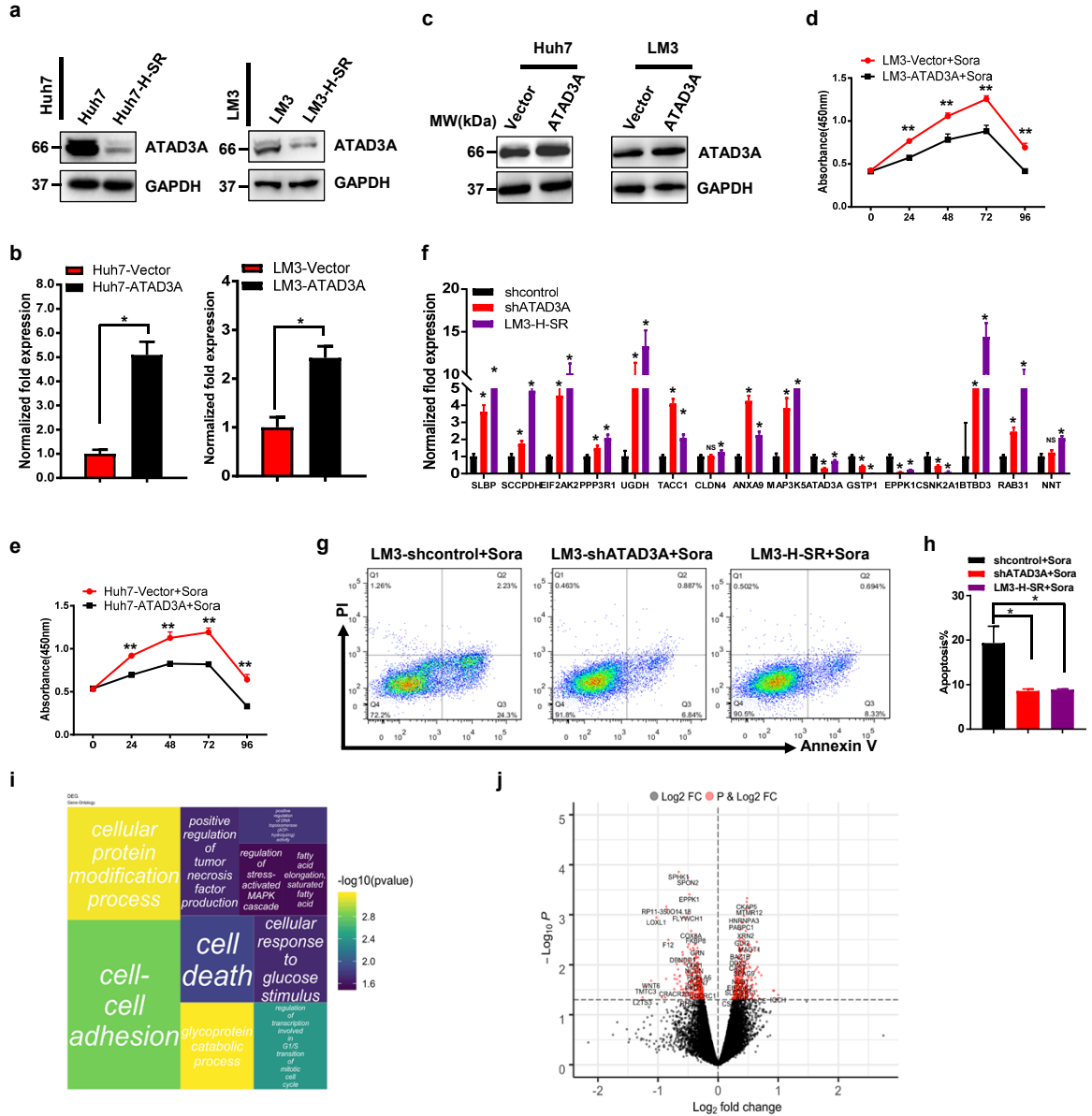

Supplement: Supplementary file 5 — Additional file 5: Supplementary Fig. 2. ATAD3A plays a vital role in sorafenib sensitivity of hepatoma cells. a Immunoblot showing expression levels of indicated proteins in control and hypoxia-induced sorafenib resistant Huh7 and LM3 cells. b qRT-PCR analysis of ATAD3A expression in LM3-vector and ATAD3A overexpression cells. c Immunoblot showing expression levels of the indicated proteins in ATAD3A overexpressing Huh7 and LM3 cells. d, e Cell viability of Huh7-vector and Huh7-ATAD3A (e) or LM3-vector and LM3-ATAD3A (d) cells under sorafenib treatment. f qRT-PCR analyses of the indicated genes in LM3-H-SR, LM3-shATAD3A and LM3-shControl. g,h Percentage of apoptotic cells in LM3-H-SR, LM3-shATAD3A and LM3-shControl cells under sorafenib treatment. Annexin V and PI respectively indicate the early and late stage apoptotic cells. i DAVID analysis of the top 10 altered pathways using KEGG in shATAD3A LM3 cells compared to control. j The volcano plots showing the differently expressed genes in shATAD3A versus shcontrol cells. Each bar represents the mean ± SEM of three independent experiments. *P < 0.05 and ** P < 0.01. [file 13046_2020_1768_MOESM5_ESM.pdf]

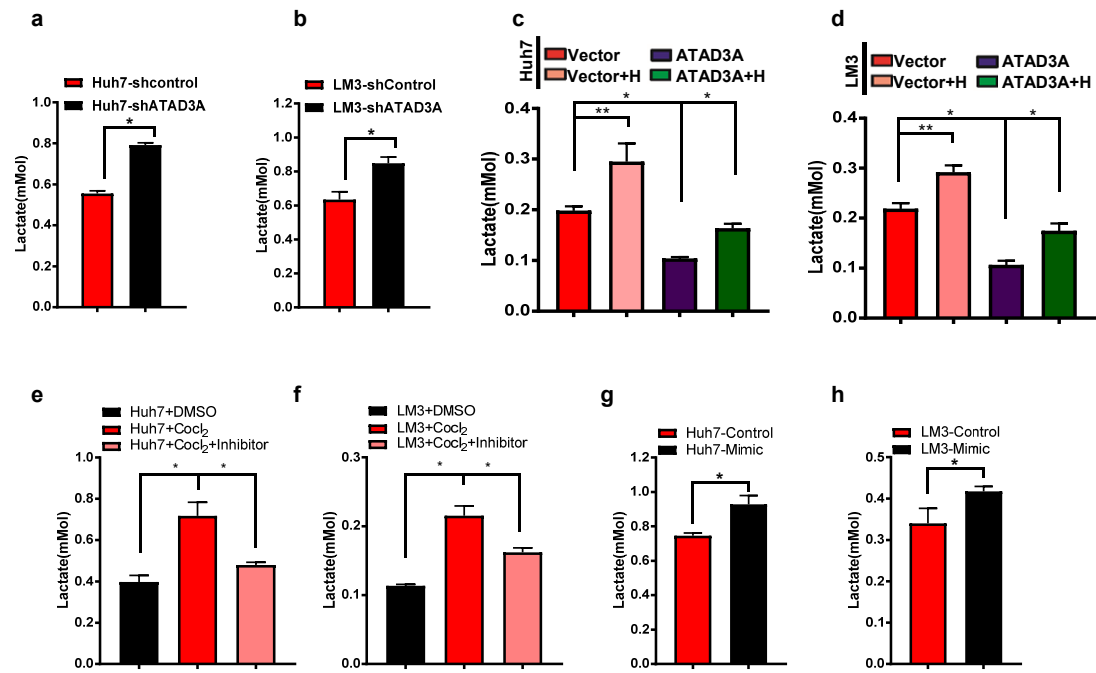

Supplement: Supplementary file 6 — Additional file 6: Supplementary Fig. 3. ATAD3A plays a vital role in lactate production of hepatoma cells. a,b Lactate levels in the supernatants of Huh7-shControl and Huh7-shATAD3A (a) or LM3- shControl and LM3-shATAD3A (b) cells. c,d Lactate levels in Huh7-Vector and Huh7-ATAD3A (c) or LM3-Vector and LM3-ATAD3A (d) cells with/without 1% O2 exposure for 24 h. e,f Lactate levels in Huh7 (e) and LM3 (f) cells with/ without miR-210-5P inhibitor transfection and with/ without CoCl2 treatment. g, h Lactate levels in control and miR-210-5P mimic-transfected Huh7 (g) and LM3 (h) cells. Each bar represents the mean ± SEM of three independent experiments. *P < 0.05 and ** P < 0.01. [file 13046_2020_1768_MOESM6_ESM.pdf]

**a**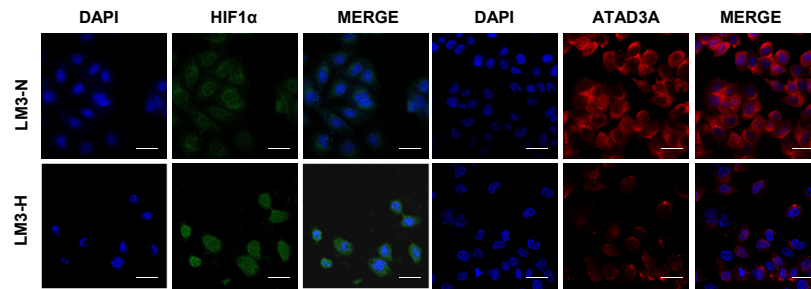**b**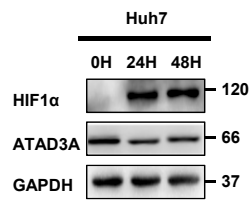**c**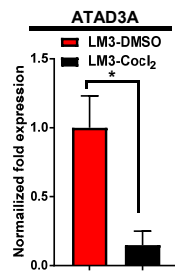**d**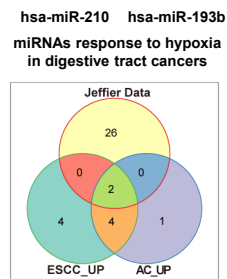**e**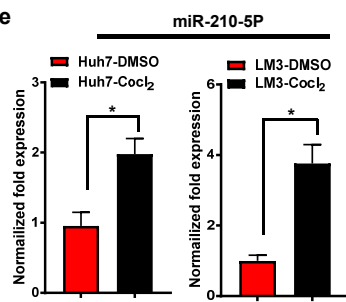**f**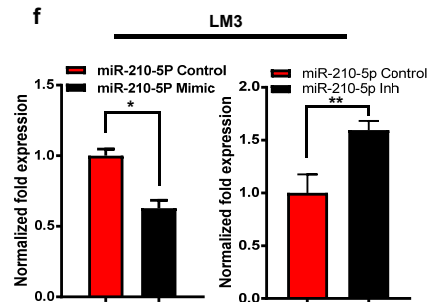

Supplement: Supplementary file 7 — Additional file 7: Supplementary Fig. 4. ATAD3A is down-regulated in hypoxia and a potential target of miR-210-5P. a Immunofluorescent staining of HIF-1α (green) and ATAD3A (red) expression in normoxic and hypoxic LM3 cells (1% O2 treatment for 24 h). Scale bar = 200 μm. b Immunoblot showing expression levels of indicated proteins in Huh7 cells with/without CoCl2 treatment for different durations. c qRT-PCR analysis of ATAD3A expression in LM3 cells with/without CoCl2 treatment. d Bioinformatics analysis of the hypoxia-responsive miRNAs from digestive cancers. e qRT-PCR analysis showing miR-210-5P levels in Huh7 and LM3 cells treated with DMSO or CoCl2 for 24 h. f qRT-PCR analysis showing the expression of ATAD3A in LM3 cells treated with miR-210-5P mimic and inhibitor. Each bar represents the mean ± SEM of three independent experiments. *P < 0.05 and ** P < 0.01. [file 13046_2020_1768_MOESM7_ESM.pdf]
